# Assessing and Improving Performance: A Longitudinal Evaluation of Priority Setting and Resource Allocation in a Canadian Health Region

**DOI:** 10.15171/ijhpm.2017.98

**Published:** 2017-08-22

**Authors:** William Hall, Neale Smith, Craig Mitton, Bonnie Urquhart, Stirling Bryan

**Affiliations:** ^1^Centre for Clinical Epidemiology & Evaluation, Vancouver Coastal Health Research Institute, Vancouver, BC, Canada.; ^2^Planning and Performance Improvement, Northern Health Authority, Prince George, BC, Canada.; ^3^School of Population and Public Health, The University of British Columbia (UBC), Vancouver, BC, Canada.

**Keywords:** Priority Setting, Evaluation, Resource Allocation

## Abstract

**Background:** In order to meet the challenges presented by increasing demand and scarcity of resources, healthcare organizations are faced with difficult decisions related to resource allocation. Tools to facilitate evaluation and improvement of these processes could enable greater transparency and more optimal distribution of resources.

**Methods:** The Resource Allocation Performance Assessment Tool (RAPAT) was implemented in a healthcare organization in British Columbia, Canada. Recommendations for improvement were delivered, and a follow up evaluation exercise was conducted to assess the trajectory of the organization’s priority setting and resource allocation (PSRA) process 2 years post the original evaluation.

**Results:** Implementation of RAPAT in the pilot organization identified strengths and weaknesses of the organization’s PSRA process at the time of the original evaluation. Strengths included the use of criteria and evidence, an ability to reallocate resources, and the involvement of frontline staff in the process. Weaknesses included training, communication, and lack of program budgeting. Although the follow up revealed a regression from a more formal PSRA process, a legacy of explicit resource allocation was reported to be providing ongoing benefit for the organization.

**Conclusion:** While past studies have taken a cross-sectional approach, this paper introduces the first longitudinal evaluation of PSRA in a healthcare organization. By including the strengths, weaknesses, and evolution of one organization’s journey, the authors’ intend that this paper will assist other healthcare leaders in meeting the challenges of allocating scarce resources.

## Background

Allocating financial resources is an essential public sector function that is performed by leaders in healthcare organizations worldwide.^1,2^ Scarcity dictates that decisions be made as to which services are funded and not funded.^3^ For example, should additional hospital resources be put into implementing an electronic medical record system or hiring support staff for an emergency department? Should more funding be allocated to research in rare diseases such as multiple sclerosis or be used for diabetes prevention programs? In essence, when there are more claims on resources than there are resources available, some form of priority setting must occur.

Despite the availability of explicit processes for priority setting and resource allocation (PSRA) based on sound economic and ethical principles (eg, accountability for reasonableness [A4R], program budgeting and marginal analysis [PBMA])*,* a 2011 survey of Canadian healthcare organizations found that 50% of respondents reported conducting priority setting primarily on the basis of historical patterns or political influence.^1^ As well, only 20% of respondents indicated that their organization conducted some form of PSRA evaluation.^4^

The lack of evaluation is perhaps not surprising given the limited number of studies that have substantively addressed the evaluation of PSRA processes.^5-7^ Ongoing application and refinement of evaluation methods is necessary to cultivate lessons and challenges faced by healthcare organizations.^5^

In order to further this work, a program of research was launched to study high performance in PSRA though case studies of Canadian organizations - and in combination with a literature review – to develop a framework of key elements for high performance.^8^ Following Donabedian’s work on healthcare quality with respect to institutional structure and organizational processes, data from the literature review and case studies were used to create the ‘High Performance Framework’ for PSRA.^8^ The framework itself includes four domains: Structures, Processes, Cultures, and Outcomes – with five elements of high performance within each domain.^8^ In the following phase of this research, the High Performance Framework was operationalized into the Resource Allocation Performance Assessment Tool (RAPAT) following a balanced scorecard approach to assessment tool development.^9-11^ The aim of RAPAT was to enable healthcare organizations to identify the strengths and weaknesses of their PSRA process in order to facilitate improvement. As such, a ‘use focused approach’ to evaluation that places the intended users’ perspective at the center of the evaluation (to ensure that action is taken as a result of the evaluation report), formed the foundation for implementation of RAPAT.^12^ In 2013, it was successfully implemented in two healthcare organizations in British Columbia, Canada. Hall et al detailed the development, application, and refinement of the RAPAT – the first documented multi-site implementation of a PSRA evaluation tool.^11^

While these previous publications have focused on the development and implementation of RAPAT itself for a methodology-focused audience, the goal of this paper is to describe the strengths and weaknesses of one pilot organization in greater depth for practitioners. Insight will also be drawn from a follow up exercise that was conducted 2 years post the original evaluation. By highlighting the achievements and challenges faced by this organization, the authors hope that this paper will serve other healthcare organizations facing resource constraints and contribute to a “set of industry best practices” as initially described by Sibbald et al.^5^

## Methods

A full description of the development and implementation of the High Performance Framework and RAPAT can be found in elsewhere.^8,11^ The focus of this methodology section will be an overview application of the tool in the pilot organization, and the subsequent follow up exercise.

### Implementation of the Evaluation Tool

During the implementation of the evaluation tool, a case methodology was followed whereby the PSRA process of the pilot organization was the bounded system that was analyzed.^13,14^ Purposive sampling was applied to select the pilot organization based on the fact that their senior management was familiar with the researchers leading this project, and that they were open to participating in this form of evaluation.

To obtain a sample that reflected the diversity within the organization, participants were selected using a purposive-criterion sampling matrix across departments, geographical locations, and levels of success with submitting past proposals for investment and disinvestment.^11^

The data collection method used semi-structured interviews that were approximately 60 minutes in duration with 45 for the evaluation and 15 minutes for participant feedback. Twenty-nine members of the organization were invited to participate and 27 were interviewed using the evaluation tool. Participants included 3 clinical leaders, 20 managers, and 4 senior managers. The interviews themselves were carried out with participants in person (n = 12), through videoconference (n = 13), and via telephone (n = 2).^11^

### Data Analysis

Analysis of the qualitative data included template analysis whereby each of the participant responses was sorted into one of the elements from the high performance framework.^15,16^ Once sorted, data were examined using content analysis to determine whether each element was a ‘strength,’ ‘area for improvement,’ or ‘weakness’ of the organization’s PSRA process.^11,17^

Content analysis was carried out in three ways. First, quotes in each element were coded as ‘positive’ or ‘negative’ based on language used. Second, examples provided in quotes served as downstream indicators for certain elements. Finally, descriptions of elements from the high performance framework were used as reference points for determining the strength of elements.

Following this analysis protocol, elements were considered strengths if the majority of participants described them in a ‘positive’ way with agreement to descriptions in the high performance framework and had supporting examples. Negatively worded quotes related to a particular element that conflicted with the description in the high performance framework were categorized as weaknesses. Elements were considered areas for improvement when their sub-elements formed a mix of strengths and weaknesses, or the negative language in the quotes was softer.

To ensure accuracy of analysis and inclusion of a broader perspective, several strength and weakness determinations were initially carried out by two research team members independently and subsequently compared to test for agreement. All finalized determinations were discussed among the core research team. More details on the analysis protocol are available elsewhere.^11^

### Follow Up Exercise

The follow up exercise was conducted 2 years post the original evaluation. Semi-structured interviews were conducted with more open-ended questions focusing on the trajectory of the organization’s PSRA process as well as strengths, weaknesses, and the future of PSRA in the organization. Participants were sampled from the original evaluation participants. Seven senior and middle managers agreed to take part in the follow up as interviewees including the chief executive officer (CEO), chief financial officer (CFO), and key members of the senior management team responsible for Finance and Budgeting. Transcripts were analyzed using the same approach as the original evaluation described above, and analysis was carried out by multiple research team members to test for agreement.

## Results

Overall, the organization’s PSRA process had many more strengths than weaknesses based on the elements of high performance in the evaluation tool. The following provides a qualitative description of their process when it was initially evaluated:

“Consultations were held to facilitate input from external stakeholders towards the creation of the organization’s strategic plan. The plan was drawn upon to create criteria for proposal assessment, and now forms the foundation of the organization-wide process. During investment and disinvestment proposal creation, managers consulted frontline staff. Short form proposals were sent to executives who decided whether a long form business case was warranted. Finance and the executive reviewed efficiency proposals, while investment/disinvestment proposals were reviewed by a working group (WG). Reports were provided to the Executive by the WG that included an independent ranking of proposals using the assessment tool. The Executive then made a decision on which proposals would be approved.”

A complete description of all strengths, weaknesses, and areas for improvement as well as the scorecard dashboard has been previously documented.^18^ Table 1 provides a dashboard of the strengths and weaknesses in the original evaluation – white cells represent strengths, grey cells represent areas for improvement, and dark grey cells represent weaknesses.

**Table 1:** Dashboard From Initial Evaluation

**Structures**	**Processes**	**Cultures**	**Outcomes**
P1 – Ability to reallocate - Strength	P1 – Process - Area for Improvement	A1 – Trust - Strength	O1 – Reallocation - Strength
S2 – Engagement - Area for Improvement	P2 – Communication - Weakness	A2 – Culture of Improvement - Strength	O2 – Endorsement - Strength
S3 – Coordination - Area for Improvement	P3 – Training - Weakness	A3 – Strategic Alignment - Strength	O3 – Understanding - Area for Improvement
S4 – Stability - Strength	P4 – Follow Through - Area for Improvement	A4 – Fit with Community - Strength	O4 – Improved Health - Strength
S5 – Time and Resources - Strength	P5 – Project Coordinator - Strength	A5 – Strong Leadership - Area for Improvement	

For the purposes of this paper, a subset of the strengths, weaknesses, and areas for improvement are presented including strengths from the initial evaluation that eventually became weaknesses in the follow up. In this way, the strengths and weaknesses presented illustrate the transformation of this organization’s process over time. In the following sections, each strength, weakness, and area for improvement element includes a short description of its importance with respect to high performance in PSRA, and is followed by results of the semi-structured interviews conducted in the pilot organization related to that element. Following the presentation of these elements, insight from the follow up exercise that was conducted two years post is presented.

### Strengths

The following elements of high performance were identified as strengths or areas for improvement in the organization, and will be presented in this section: Criteria and Assessment Tool, Frontline Staff Involvement, Ability and Authority to Move Resources.

#### Process - Criteria and Assessment tool

If mechanisms employed to guide resource allocation are inequitable or even non-explicit, the distribution of resources will potentially be sub-optimal and will certainly lack transparency.^3,19^ Within these mechanisms, the criteria used to evaluate proposals are likely to have profound implications on the final decisions made.^19^ By linking criteria to the strategic plan of the organization and consistently applying them to each proposal using a formal assessment tool, organizations can better direct their resources to high priority areas that will meet their long-term goals and objectives.^15^

Prior to the implementation of their PSRA process, managers in the pilot organization reported that “*there [were] no objective criteria*” used in the resource allocation process. “*It was basically whoever could yell the loudest*” (Middle Manager). As part of introducing a more formal approach to resource allocation, this organization developed criteria “*tied to [its] strategic plan*” (Senior Manager).

To ensure consistency in applying the criteria, the test organization developed an assessment tool based on the criteria and applied this to its funding decisions. “*The criteria are explicitly stated in the process and are part of the formal ranking tool that applies to every submission*” (Senior Manager). Further, within the assessment tool, criteria were defined and weighted, and scoring guidelines were provided to users. In addition to self-scoring by the proposal authors, a validation group (consisting of mid-level managers and clinicians) also evaluated and scored each proposal using the assessment tool. In this way, a ‘peer-review check’ encouraged validity and consistency in scoring.

#### Frontline Staff Engagement

By soliciting proposals for investment and disinvestment from the frontline, organizations can engage staff in PSRA decisions. In doing so, they can take advantage of direct experience to deliver more accurate assessments of proposals, and potentially improve buy-in to final resource allocation decisions.^15^

In the pilot organization, staff participation in the PSRA process was evident in several ways. The most common method appeared to be during the creation and refinement of proposals.

*“You may have a working proposal and it’s very much then refined by discussion with staff as to feasibility. You also need to operationalize that plan. And obviously the input from staff is really important to be able to do that”* (Middle Manager).

Even in the creation of funding proposals, managers reported that staff were very helpful with suggestions. For example, “*We don’t need this extra person on this four hours*,” or “*we continue to throw away a third of this because it doesn’t get used before it’s outdated*” or, “*you buy all these things and it’s really expensive and we should be going back to these because they’re cheaper and they’re just as good*” (Middle Manager). This “*more open and transparent*” approach was very different from previous years when managers “*had 48 hours to come up with ways to cut 2, 3, 4%*” arbitrarily from their budget and “*kept it under wraps until [they] were ready to move*” (Middle Manager).

#### Ability and Authority to Move Resources

Maximizing the benefits of health services offered to an entire population requires re-allocation of resources from low yield programs to higher yield programs.^3^ As Smith et al argue, “If the senior management team is constrained in its ability to make these organization-wide trade offs, optimal distribution of resources may not be achieved.”^15^ This key outcome of PSRA processes is addressed in both the structure and outcome domains of the evaluation tool and high performance framework.

Many senior managers in the pilot organization agreed that they had the technical capacity to re-allocate resources and that there “*is still the availability to move money around and make resource allocation decisions*” (Senior Manager). Some acknowledged that before they make a decision they have to “*make sure that it aligns with government’s vision and change agenda*” (Senior Manager). As a result, their authority to re-allocate resources may be somewhat limited. Despite these limitations, all of the senior managers interviewed reported that re-allocations of resources took place during their last PSRA cycle.

In the following section, a subset of the weaknesses from the pilot organization’s PSRA process will be presented based on the original evaluation.

### Weaknesses

The following elements of high performance were identified as weaknesses or areas for improvement in the organization, and will be presented in this section: Training and Education, Communication, Timelines and Deadlines, Program Budgeting.

#### Training and Education

Without a formalized education program for stakeholders, organizations may find difficulty engaging their staff members and implementing a PSRA process.^20,21^ Studies of past implementations have revealed that education is very much needed to ensure understanding and build acceptance.^16^

Since the PSRA process was relatively new to the organization at the time of the evaluation, “*there’s a bit of a learning curve there for everybody*” (Middle Manager). However, “*new staff orientation is definitely a weakness*” for the organization (Middle Manager). Currently, a learning-on-the-job approach is common whereby managers are told that “*this is the process and here is the document... fill it in*” with minimal or no prior training given (Middle Manager).

Senior management agreed that no formal PSRA education exists for staff in the organization, and that “*most front-line staff would not know exactly how resource decisions get made*” (Senior Manager). Tellingly, mid-level managers also reported confusion about the process, and described lower level management and staff that were uncertain even when it came to the name of the priority setting process – “*I’m not always sure still when I say PBMA to my management group on the ground here that they all know exactly what I’m talking about*” (Middle Manager). This lack of understanding was clearly demonstrated when one of the mid-level managers interviewed was completely unaware of key aspects of the process including the use of a criteria-based assessment tool to score and rank proposals (Middle Manager). One senior manager further iterated this: *“Staff, no, I don’t think our staff really understands the PBMA process”* (Senior Manager).

Participants also recognized a lack of training as a risk to the integrity and effectiveness of their PSRA process. Without an understanding of their own organization’s process, frontline managers and staff are likely to “*lose confidence in the process*” (Middle Manager), create “*proposals that are not strategically oriented at all*” (Senior Manager), and “*game the system*” (Middle Manager).

#### Timelines and Communication

Effective communication is essential throughout the entirety of a PSRA process. This includes ensuring that the criteria used to make decisions, the decisions themselves, and the implementation plans are well understood.^15^ In the event of proposal rejection, highlighting the rationale for decisions will also enable staff to understand why they did not get funding and may strengthen future attempts.^20^

For the pilot organization, a lack of communication during and after their PSRA process was identified as a significant weakness in their process. Middle managers reported general uncertainly with the process, and how they should proceed with proposal submission. *“The messaging is so confusing at times that it’s not like you know exactly what is required of you and how to move forward”* (Middle Manager).

Lack of clarity had reportedly led to the creation of proposals that are not aligned with the strategic priorities or criteria of the organization.

*“What we could be stronger at is actually linking the proposals to the criteria... We’ve got a strategic evaluator, a strategic executive, looking at it. But we’re looking at a bunch of proposals that are or are not sort of strategically oriented at all”* (Senior Manager).

Members of the pilot organization also agreed that “*sometimes we have trouble keeping on our timeline target*” and “*there’s often not enough time or the time is poorly communicated*” (Middle Manager). Issues with the timeline were reported even in the preparation stages of the process. Discussions around potential proposals were reportedly occurring “*in the summer when a lot of our [frontline] managers aren’t available*” forcing decisions to be made without their input (Middle Manager). Lack of clarity and time restrictions also prevented managers from engaging with their staff.

*“I think we just have to attach guidelines to the steps and that might really support getting an early start and having time to actually listen to the staff. It’s not that managers don’t want to ask or engage or hear from their staff. I think we just set up really tight timelines and there’s no time to do it”* (Middle Manager).

This weakness extended to communicating the rationale for resource allocation decisions as well with middle managers not receiving “*communications back from the committee*” after they had submitted a proposal (Middle Manager). Without this feedback, managers reported being uncertain as to whether they should pursue proposals further and feeling less engaged and distrustful of the entire process.

#### Process - Program Budgeting

In order to facilitate explicit comparison of services, a map of current activity and expenditure is recommended. Program budgets document how resources are being spent within organizations, and can be used as a starting point for managers to identify high and low priority programs in their respective portfolios.^3^ To create a program budget, activity and cost data from each service must be accessible through administrative data or be collected prospectively. High level costing will generally suffice since fine precision is not the aim of program budgeting.^3,6^ Once an overview of resource expenditure has been collated, organizational criteria can be applied to each service to create a ranking of programs. Low priority services or aspects of services can then become options for disinvestments.

Despite operationalizing their strategic plan in the form of weighted criteria applied consistently to proposals using an assessment tool, the pilot organization has not trained or educated its managers to perform reviews of their portfolios so that they might be able to better identify low and high priority services. Both middle and senior managers reported that significant variation existed in the extent to which different portfolios map their services since it is performed at the “*discretion*” of individuals, and is often not applied “*the way it is supposed to be*” in a comprehensive manner (Middle Manager).

They also described the task of creating a program budget for their portfolios as “*daunting,*” “*overwhelming,*” and “*definitely not something that could be done off the side of one’s desk*” (Senior Manager) (Middle Manager). As a result, rather than performing a comprehensive review of their portfolio and identifying low and high priority services, managers admitted to very informal processes for determining which proposals were put forward for disinvestment.

*“Okay so this actually is sort of rising to the top let’s put forward this and nobody’s really utilizing this or the service is not aligning well so let’s score that one and see if we disinvest”* (Middle Manager).

Although this approach has been passable in previous cycles, both middle and senior managers acknowledged that a “*comprehensive review [of portfolios] is needed*” because “*next year [they] will have skinned off all of that low-hanging fruit*” (Middle Manager), and without a “*firmer framework*” for mapping and ranking portfolios, disinvestments will continue to be “*more in the order of efficiencies rather than... the lowest priority programs*” (Senior Manager).

In the following section, insights for the follow up exercise that was conducted two years post this original evaluation will be presented.

### Results - Follow Up Exercise

The follow up exercise revealed a dramatic shift in PSRA within the pilot organization. Table 2 illustrates the strengths and weaknesses in the follow up evaluation of the organization’s PSRA process - white cells represent strengths, grey cells represent areas for improvement, and dark grey cells represent weaknesses.

**Table 2:** Dashboard From Follow Up Evaluation

**Structures**	**Processes**	**Cultures**	**Outcomes**
P1 – Ability to reallocate - Strength	P1 – Process - Area for Improvement	A1 – Trust - Strength	O1 – Reallocation - Strength
S2 – Engagement - Area for Improvement	P2 – Communication - Weakness	A2 – Culture of Improvement - Strength	O2 – Endorsement - Strength
S3 – Coordination - Area for Improvement	P3 – Training - Weakness	A3 – Strategic Alignment - Strength	O3 – Understanding - Area for Improvement
S4 – Stability - Strength	P4 – Follow Through - Area for Improvement	A4 – Fit with Community - Strength	O4 – Improved Health - Strength
S5 – Time and Resources - Strength	P5 – Project Coordinator - Strength	A5 – Strong Leadership - Area for Improvement	

Participants reported that the formal process in place during the time of the original evaluation had been stopped. Departments were no longer required to submit proposals for investment or disinvestment that were scored using a set of criteria. While some managers felt that the process had “*deteriorated*” or “*eroded a little bit,*” others viewed this step back as necessary to avoid a situation where “*expectations [are created] that we can’t deliver on*” (Middle Manager) (Senior Manager).

This decision resulted in forfeiture of the organization’s prior strength of utilizing criteria and the assessment tool as well as their ability to re-allocate resources across departments. There was also recognition that some of the weaknesses identified in the original evaluation had continued including a lack “*of outward visibility of what is going on”* and a paucity of training opportunities for managers *“in terms of reading your budget… how to do resource allocation within your budget. This is fairly informal education that happens”* (Middle Manager).

Rather than true disinvestment proposals being put forward (ie, those that would incrementally reduce service), managers recognized that their process had been delivering efficiencies (ie, delivering the same level of service with less resources). This was largely attributed to their lack of comprehensive efforts around program budgeting and service planning when the organization wide PSRA process was being implemented.

*“Because if you don’t do this piece [program budgeting and service planning] then all you end up with are efficiencies – whatever is safe: painting buildings, and maintenance stuff”* (Senior Manager).

Multiple reasons for the discontinuation of the organization wide PSRA process and the perpetuation of these weaknesses were suggested including:

- A loss of sponsorship due to the complexity of an organization - *“We allow ourselves to excuse ourselves when things are complex. Complexity is too easy an excuse to sort of say: “there was a lot of work behind that [organization wide PSRA process]. Maybe there is an easier way”* (Middle Manager).
- A lack of external fiscal pressure - *“Our financial picture over the past couple of years has not been too bad. So there hasn’t been that burning platform”* (Middle Manager).
- No new money for investment - *“The budget from the province has been tighter as we expected it to be. So I’m thinking that there was less interest in doing only reductions with [the organization wide PSRA process] when there wasn’t a lot for re-investment”* (Senior Manager).
- A lack of foundational understanding with respect to the programs and services offered - *“To me it’s clear that we need a service model so that when you are looking at [the organization wide PSRA process] it’s more “what are the things we’re offering.” Then you would identify the resources, the priorities, the outcome implications on a marginal basis – and then make prioritization decision”* (Senior Manager).

Despite this shift away from an organization wide PSRA process, respondents did report some positive developments from implementing a more formal process. Examples included use of evidence and data including “*outcomes and hours per patient day”* (Middle Manager) to evaluate programs as well as a longer-term perspective by “*projecting out longer distances like 5 years out*” (Senior Manager). From a cultural perspective, respondents reported that the principles of resource scarcity - “*there is no new money*” - and opportunity cost - “*if they want to do something new, they need to find it in their current budget” -* seem to have filtered down to the middle and frontline levels of the organization as well (Senior Manager).

*“People understand that the [resource allocation] processes are more fair and equitable now. They came to the meeting to speak to their needs, but also we are coming to listen to others needs recognizing that decisions will be based on the greatest amount of information. To me, that was a really big improvement and that that was due to people’s experience with the [organization wide PSRA] stuff”* (Senior Manager).

When questioned about the future of their approach to PSRA, participants agreed that their previous organizational process allowed them to balance their budget in times of austerity; however, new challenges would require a more comprehensive approach:

*“We are going to be in a challenge where the money from outside is fixed, there are no more inefficiencies, but there is demand for new programs. So how do we fund those, and compare those new programs with our lower priority programs?”* (Senior Manager).

In order to address these upcoming challenges and achieve true re-allocation of resources from low to high priority areas, participants suggested that a more in-depth understanding of their services and programs was necessary. Once this foundation of understanding had been created, they anticipated that a return to an organization wide PSRA would be possible, and that their “*experience with the PSRA is going to come in really handy – because, you know, we are going to be faced with some difficult decisions”* (Middle Manager).

*“I’m thinking that as we get farther along, and we get more information on what services are needed… that we take a look at [a organization wide PSRA process] again. Because we will need to shift resources over time”* (Senior Manager).

## Discussion

To date, evaluations of PSRA within healthcare organizations have followed a cross-sectional approach.^5-7^ This study represents the first published longitudinal evaluation of PSRA within a Canadian healthcare organization. By presenting results from evaluations conducted two years apart, this paper demonstrates how the strengths and weaknesses of an organization’s PSRA process can dramatically change over a relatively short period of time.

The initial evaluation identified strengths of the organization’s PSRA process including a comprehensive approach that assessed proposals for investment and disinvestment using criteria that ultimately enabled the re-allocation of resources across departments. This evaluation also revealed weaknesses of the process including a lack of program budgeting, training for managers, and communication. Two years after the original evaluation, the follow up found that a significant shift had taken place. The pilot organization was no longer conducting their comprehensive PSRA process, and in doing so forfeited several of their key strengths.

Given this dramatic shift, the factors underlying that 2-year time period are of particular interest. Internal forces reportedly included a lack of understanding with respect to programs and services as well as a loss of sponsorship, while external forces reportedly included a lack of new money for investment and a decrease in fiscal pressure.

Although the shift in approach to PSRA was substantial, the impact of the internal forces could have been predicted by the original evaluation that identified a lack of program budgeting as a weakness in the organization. A thorough understanding of the programs and services that an organization delivers as well as the costs and outcomes of those services using a program budget is crucial to effective resource allocation.^3,17^ Despite this importance, Public Health was the only department in the pilot organization that had created a comprehensive program budget. Since the majority of managers did not have this map of their programs, they were unable to deliver true disinvestment proposals and instead proposed efficiencies in their place.

The impact of external forces may have also been predicted. In budget theory literature, two models dominate: *Incrementalism* – marginal increases or decreases to budgets year over year that are often evenly distributed equally to departments and *Rationalism* – resource allocation decisions made on the basis of an agreed upon set of goals or vision.^22^ Incrementalism is generally favoured during times of stability since it provides a straight forward method of allocating resources, does not require very much capacity, and is viewed as ‘fair’ by participants since each department is treated equally.^22^ Conversely, times of economic turmoil and decreased revenues generally force organizations to more carefully consider their resource allocations using a more ‘rationalistic’ approach.^22^ In the case of the pilot organization, decreases in revenue from the provincial government preceding the original evaluation likely prompted the application of a stronger, more comprehensive PSRA process. Once the revenue from the provincial government stabilized, a return to a more incremental approach to PSRA took place.

While a regression in the approach to PSRA has certainly taken place in the pilot organization, there was recognition of future external forces including greater demand from an aging population and more expensive treatments that will contribute to a financial environment that will drive a greater need for a more rational process. To prepare for this transition, respondents reported efforts to address the lack of program budgeting and service planning in their organization. In this way, they have made a commitment to address the internal factors that prevented them from continuing their organization-wide PSRA process in order to address upcoming challenges that will pose external pressure.

The exploratory nature of this research includes several important limitations. Firstly, although every individual from the original evaluation group of 27 was invited to participate in the follow up evaluation, only seven were available. Although these seven included the CEO, CFO, and key senior managers responsible for Budgeting and Finance in the organization, an ideal follow up would have included a larger sample. Given the 2-year gap between the original evaluation and follow up, and the fact that no participants responded with a rejection of the invitation, it is possible that some of the individuals from the original sample were no longer in the same positions or working within the organization itself. The homogeneity of responses and positions of the seven members that were interviewed leads us to believe that the results have strong internal validity; however, as previously stated, a larger sample would lend greater credibility to these findings. This may be achieved in future applications of RAPAT through online assessment. Secondly, generalizability of results may be limited due to an n = 1 in this study. Indeed, this pilot organization served as the first case study of a longitudinal PSRA evaluation. In order to facilitate reproducibility and external validity of findings, greater systemization of PSRA evaluation must be introduced – one method to achieve this could be through online delivery and accreditation of organizations.^23^

## Conclusion

As healthcare budgets continue to stagnate while demands for services grow, the need for explicit high performing PSRA processes increases. Evaluating the strengths and weaknesses in these processes is critical, and yet relatively few research efforts have been aimed at addressing this issue. This paper represents the first longitudinal application of the RAPAT. In the initial application, strengths and weaknesses of the pilot organization’s process were identified. A follow up exercise two years later revealed a significant shift away from their previous process due to a variety of internal and external factors. By documenting this shift, this paper highlights the importance of longitudinal analysis in the area of PSRA, and identifies new areas of study including the sustainability of PSRA processes, and the role of internal and external forces in shaping an organization’s approach.

## Acknowledgements

The authors would like to acknowledge the staff, clinicians, and senior management from the health region that participated in the evaluation. Funding for this research was provided by the Canadian Institutes for Health Research (CIHR) through a Partnership for Health Systems Improvement (PHSI) grant.

## Ethical issues

Ethics for the study was obtained from the University of British Columbia Research Ethics Office, Canada.

## Competing interests

Authors declare that they have no competing interests.

## Authors’ contributions

WH conducted interviews, analyzed data, and wrote first manuscript. NS assisted with interviews, assisted with data analysis, and assisted with manuscript writing and editing. CM as principle investigator on original evaluation project, assisted with manuscript writing and editing. BU coordinated interviews, assisted with manuscript writing and editing. SB assisted with manuscript writing and editing.

## Authors’ affiliations

^1^Centre for Clinical Epidemiology & Evaluation, Vancouver Coastal Health Research Institute, Vancouver, BC, Canada. ^2^Planning and Performance Improvement, Northern Health Authority, Prince George, BC, Canada. ^3^School of Population and Public Health, The University of British Columbia (UBC), Vancouver, BC, Canada.

## Key messages

**Implications for policy makers**

- Guidance on how to manage the challenges of resource scarcity that are becoming increasingly prevalent in our current economic environment.
- Lessons related to the strengths and weaknesses of another organization’s process for priority setting and resource allocation (PSRA).
- Documentation of the implementation of an evaluation tool that could facilitate assessment and improvement of PSRA processes.
- Promote awareness of external factors that can impact an organization’s PSRA process.

**Implications for public**

As payers and users of healthcare, the public should be deeply concerned about the processes used to set priorities and allocate resources. Effectively, these processes could determine whether our loved ones receive a particular chemotherapy drug, or whether they have access to a primary care physician, or how long they wait for a knee surgery.

Given the importance of these processes, it is vital that we critically examine and evaluate them to ensure best practices are being implemented and that our society’s values are being reflected in the decisions that are made. This paper documents the first implementation of an evaluation tool (RAPAT – resource allocation performance assessment tool) to evaluate a healthcare organization’s process over a three-year period.
